# Portable paper-based electrochemiluminescence test incorporating lateral-flow immunosensors for detection of interferon-γ levels

**DOI:** 10.3389/fbioe.2023.1131840

**Published:** 2023-02-07

**Authors:** Shichao Yuan, Guihua Xie, Xiang Yang, Yu Chen, Hongbin Zhang

**Affiliations:** ^1^ Department of Basic Medical Research, General Hospital of Southern Theater Command of PLA, Guangzhou, China; ^2^ Guangzhou Leide Biotechnology Co, Ltd, Guangzhou, China

**Keywords:** electrochemiluminescence, interferon-γ, lateral flow immunosensors, paper-based, POCT (point-of-care testing)

## Abstract

Tuberculosis (TB) poses a serious threat to human health and social development. Accurate diagnosis of *mycobacterium tuberculosis* infection plays a critical role in the prevention and treatment of tuberculosis. Interferon-γ (INF-γ) release assay (IGRA) is currently the only quantitative tuberculosis infection diagnosis method. An accurate, fast, and easily handled INF-γ detection method is the key to obtaining accurate results. Herein, we report a novel paper-based electrochemiluminescence (ECL) method based on lateral flow immunosensors that combines the easy handling characteristics of immunochromatography and the high sensitivity of electrochemiluminescence to detect IFN-γ. To our knowledge this is the first INF-γ detection method that combines immunochromatography with electrochemiluminescence. The paper-based ECL-LFI test consists of a sample pad, conjugation pad (with binding antibody IFN-γ-Ab1 conjugated with ruthenium tripyridine), detection pad (with capture antibody IFN-γ-Ab2 immobilized on nanospheres), absorbent pad, and electrode for signal activation. The ECL signal is obtained by cyclic voltammetry scanning at a speed of 0.1 V/s in the detection area of the paper-based ECL-LFI test. In our experiments, the paper-based ECL-LFI test exhibited a minimum detection limit of 2.57 pg/mL within 12 min, and a broad detection range of 2.57–5,000 pg/mL, with repeatability of 8.10% and stability of 4.97%. With the advantage of high accuracy and sensitivity, easy handling, and low user training requirements, this ECL-LFI test might be used as point-of-care testing (POCT) in the IGRA for tuberculosis diagnosis.

## 1 Introduction

Interferon gamma (IFN-γ) is a major immunoregulatory cytokine predominantly produced by T cells and innate lymphoid cells (Petermann et al., 2019). IFN-γ not only has antiviral, anti-microbial infection, antitumor, and other biological functions, but also plays a crucial role in innate immunity and acquired immunity (Hawerkamp et al., 2020; Thibaut et al., 2020; Caputa et al., 2022). It can also be used to detect immune levels or certain diseases, such as tuberculosis (TB) (Bergamaschi et al., 2021; Hlaca et al., 2022).

The IFNB-γ release assay (IGRA) method is commonly used in the diagnosis of TB infection (Sloot et al., 2020). The traditional IGRA method requires professional operation, instruments, and experimental platforms, and the process is time-consuming and laborious (Hamada et al., 2021; Petnak et al., 2022). Therefore, an accurate, fast point-of-care testing (POCT) method for the IGRA is urgently needed.

Electrochemiluminescence (ECL) sensors incorporate a combination of electrochemistry and measurement of visual luminescence, and have the advantages of being highly sensitive, non-hazardous, and inexpensive; moreover, they exhibit a linear response over a wide range (Zhang and Liu, 2020; Ahmadi et al., 2021; Rizzo et al., 2021). Several electrochemical luminescence immunoassays have been established to detect tuberculosis markers Lipoarabinomannan (LAM), ESAT-6, IFN-γ and Interleukin (IL-2) (Zhou et al., 2017a; Zhou et al., 2017b; Sigal et al., 2018; Broger et al., 2019). The luminous substances emit light only under electrical stimulation, which reduces the sensitivity of ECL to interference by external factors (Bauer et al., 2021). Several reported ECL assays for IFN-γ adopt only paper-based circular detection (Sennikov et al., 2003; Li et al., 2020). The lateral-flow immunoassay (LFI) is a paper-based platform that is inexpensive, simple to perform, rapid, and portable, and thus is ideal for use in point-of-care (POC).

In this report, a lateral flow immunosensor combined with ECL was developed to detect IFN-γ. Nanospheres combined with capture antibodies were adopted to improve the capture efficiency and ECL intensity. The entire testing process takes only 12mins.The ECL-LFI adopts dry chemical diagnosis, which is different from traditional wet chemical diagnosis. Moreover, there is no need to add samples many times, samples can be added at once to detect the IFN-γ concentration without other operations, which is highly desirable for new and sudden infectious diseases. The combination of paper and screen printed electrode (SPE) makes ECL-LFI portable and mass producible. Moreover, the RPEL-B POCT electrochemical luminescence detector we used is close to a notebook, and the weight is only 1 kg, which is highly conducive to application in remote areas with poor medical conditions.

## 2 Materials and methods

### 2.1 Materials

IFN-γ-Ab1, IFN-γ-Ab2, and antigen were purchased from Guangzhou Leide Biotechnology Co., Ltd. (China). N-Hydroxy succinimide (NHS), 1-(3-Dimethylaminopropyl)-3-ethylcarbodiimide hydro (EDC), Ru (bpy)_2_ (mcbpy-O-Su-ester) (PF_6_)_2_ (Ru-NHS ester), Butyldiethanolamine (BDEA), Tripropylamine (TPA), and TWEEN-20 were purchased from Sigma–Aldrich (United States of America). Elecsys buffer was purchased from Roche (Switzerland). Nanospheres (200-nm diameter) were purchased from Biotron Biotechnology Co., Ltd. (China). Centrifugal tubes (15 mL) and 5-mL 7K MWCO Zeba™ spin desalting columns were purchased from Thermo Fisher Scientific (United States of America). A Three Screen Printed Electrodes (SPEs) were purchased from Zensor Technology (China). Glass fiber RB-45 and absorbent paper ABP-S270 were purchased from Shanghai KingBio Biotechnology (China). Whatman No. One and Whatman No. 102 filters were purchased from Whatman (United States of America). PT-0.45 μm was purchased from Cytiva (United States of America). Biodyne C was purchased from PALL (United States of America). Heterophilic antibody blocker (HBR) and dimethyl sulfoxide (DMSO) were purchased from Bio Liye Technology (China).

### 2.2 Instruments

A RPEL-B POCT electrochemical luminescence detector was purchased from Xi’an Remex Analysis Instruments (China). An HGS210 high-speed cutting machine and HGS510-1 bench-type gold spraying marking machine were purchased from Hangzhou AutoKun Technology (China). A rotary disc mixer was purchased from Aquapro International Co. (United States of America).

### 2.3 Preparation of antibody-functionalized ECL nanoprobes

**FIGURE 1:**
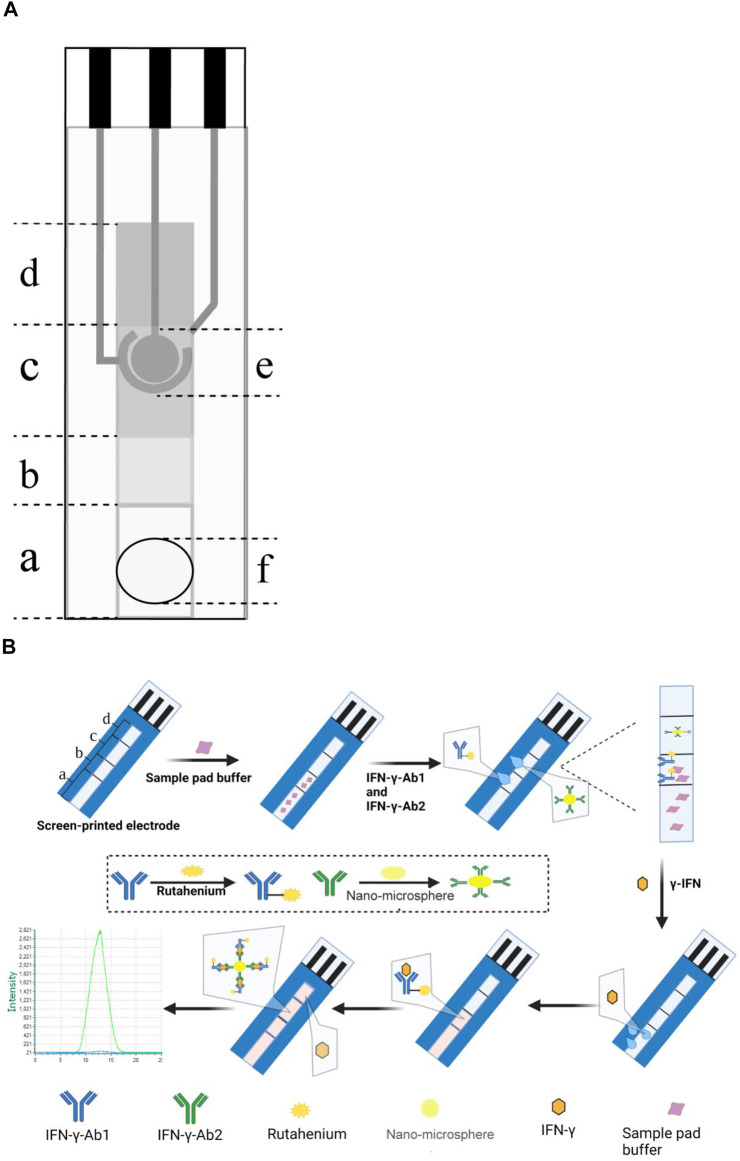
**(A)** Schematic diagram of the three-electrode SPE ECL-LFI; **(a)** Sample pad, **(b)** conjugation pad, **(c)** detection pad, **(d)** absorbent pad, **(e)** electrodes (reference electrode, working electrode, and counter electrode), and **(f)** sample loading hole. **(B)** Illustration of the ECL-LFI for detection of IFN-γ; **(a)** sample pad, **(b)** conjugation pad, **(c)** detection pad, and **(d)** absorbent pad.

The method of labeling the antibody with Ru-NHS ester was based on a previously reported method with some modifications (Zhou et al., 2014). First, 1 mg of Ru-NHS ester was dissolved in DMSO to 10 mmol/L and then diluted with distilled water to 1 mmol/L. IFN-γ-Ab1 was diluted with phosphate buffered saline (PBS) to 25 μmol/L. Second, 500 μL of antibody solution and 1,000 μL, 500 μL, and 250 μL of Ru-NHS ester solution were mixed in separate samples; the molar ratio of Ru-NHS ester to antibody was 80:1, 40:1, and 20:1, respectively. The samples were then wrapped with tin foil and rotated on the rotary disc mixer for 12 h. Following the reaction, a 5-mL 7K MWCO Zeba™ spin desalting column was used to remove free Ru-NHS ester from the Ru-labeled IFN-γ-Ab1 (labeled antibodies). See Figure 1B.

Preparation of captured antibodies IFN-γ-Ab2 coupled nanospheres was performed as shown in Figure 1B.

Diluted capture antibodies was added to activated microspheres and then blocked with 0.05-M Tris-HCl and 1% Bovine albumin (BSA) before being resuspended in a resuspend solution.

### 2.4 Preparation of immunochromatographic strips

To determine the paper suitable for detection, different papers were cut into several discs with a diameter of 6 mm. Each disc was placed in the circular detection pad of the three-electrode SPE. Labeled antibodies was diluted to 0.2 mg/mL according to the method described in Section 2.3 and mixed with Elecsys buffer. Subsequently, 6 µL of diluted antibody was added to the disc for the electrochemiluminescence test.

The captured antibodies was sprayed on the detection pad using the HGS510-1 bench-type gold spraying marking machine. Following that, a 2% sucrose solution was added to protect the antibody adsorbed on the paper.

Each sample pad was pretreated with 0.05 M Tris (pH 7.4), 1% BSA, 0.5% sodium casein, 0.5% trehalose, 2% sucrose, 0.5 mg/mL HBR, and 0.9% NaCl. Following overnight drying in an oven at 37°C, the labeled antibodies was sprayed on the bonding pad with the HGS510-1, followed by oven drying again. The labeled antibodies was sprayed onto the conjugation pad using the HGS510-1 machine and then oven-dried again.

The entire paper-based channel is 7 cm long and 6 mm wide. The sample pad is 2.5 cm long. The conjugation pad is 1.0 cm long. The detection pad is 1.5 cm long, and the absorbent pad is 2 cm long (Figure 1A).

### 2.5 ECL detection of IFN-γ

**FIGURE 2:**
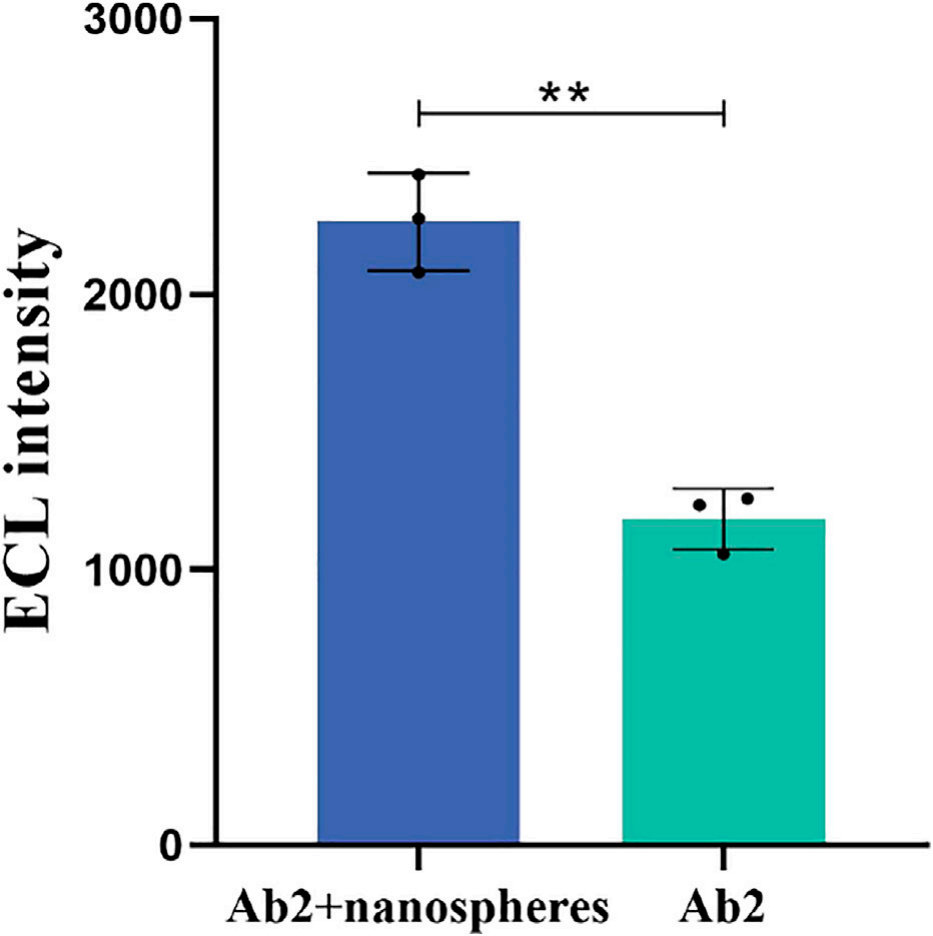
Effect of IFN-γ-Ab2 coupled nanospheres on ECL intensity. The concentration of IFN-γ is 200 pg/mL *p* < 0.05 was considered significant; *p* < 0.01.

The immunoassay was based on the double-antibody sandwich method. To begin, the sample was diluted with 0.1-M PBS (pH 7.4) and 50 μL Elecsys buffer to a final volume of 150 μL. The diluted sample was then dropped into the loading hole of the paper-based ECL-LFI. Through optimization of reaction time, following a reaction in the dark for 12 min, ECL detection was carried out as shown in Figure 2. Potential scanning was performed by cyclic voltammetry scanning from 0.2 to 1.5 V; the photomultiplier tube voltage was set at 800 V.

### 2.6 Statistical analysis

Standard deviations were determined by relative expression ratios of three replicates for each measurement. In the case of a normal distribution, a *t*-test or analysis of variance (ANOVA) test with Bonferonni correction was applied. A *p*-value less than 0.05 was considered significant (*p < 0.05; **p < 0.01). All statistical analyses were performed using SPSS 21.0 software (SPSS Inc., United States of America).

## 3 Results and discussion

### 3.1 Characterization of antibody-functionalized ECL nanoprobes

The OD value of the ECL nanoprobe was measured with a spectrophotometer. According to the formula [Label ratio] = A_antibody absorption_×dilution factor/13700 M^−1^cm^−1^×C_antibody concentration_ (Zhou et al., 2014)_,_ the labeling rates of the 20:1,40:1, and 80:1 Ru-NHS ester to antibody molar ratios were 45.5%, 53.1%, and 44.8%, respectively. Thus, the labeling rate is the highest when the molar ratio is 40:1.

RPEL-B was used to measure the effect of IFN-γ-Ab2 coupled to nanospheres. The average ECL intensity of IFN-γ-Ab2 coupled with nanospheres and IFN-γ-Ab2 was 2,264 and 1,184, respectively (n = 3). Figure 2A presents a comparison with the spraying of detection antibody IFN-γ-Ab2 alone; a higher ECL intensity was obtained after IFN-γ-Ab2 was bound to the nanospheres and then sprayed onto the detecting area of the strip; the difference was statistically significant (*p* < 0.01).

The ECL intensity of the materials used in the ECL-LFI test was detected to eliminate the signal interference to the Ru-NHS ester. The average ECL intensity of Ru-NHS ester, IFN-γ, IFN-γ-Ab1, IFN-γ-Ab2, nanospheres, and Elecsys buffer are 4,158.7, 79.3, 62.3, 79.8, and 113.3, respectively (n = 3). Figure 2B illustrates that the materials used in ECL-LFI do not interfere with the ECL intensity of Ru-NHS ester (*p* < 0.01).

### 3.2 Effect of co-reactants on ECL intensity

In this study we optimized several crucial parameters of the reaction of co-reactants with Ru-NHS ester. The experiment was carried out on Whatman No. 1 filter medium, and the blank group was 6 µL of 0.2 mg/mL labeled antibodies.

**FIGURE 3:**
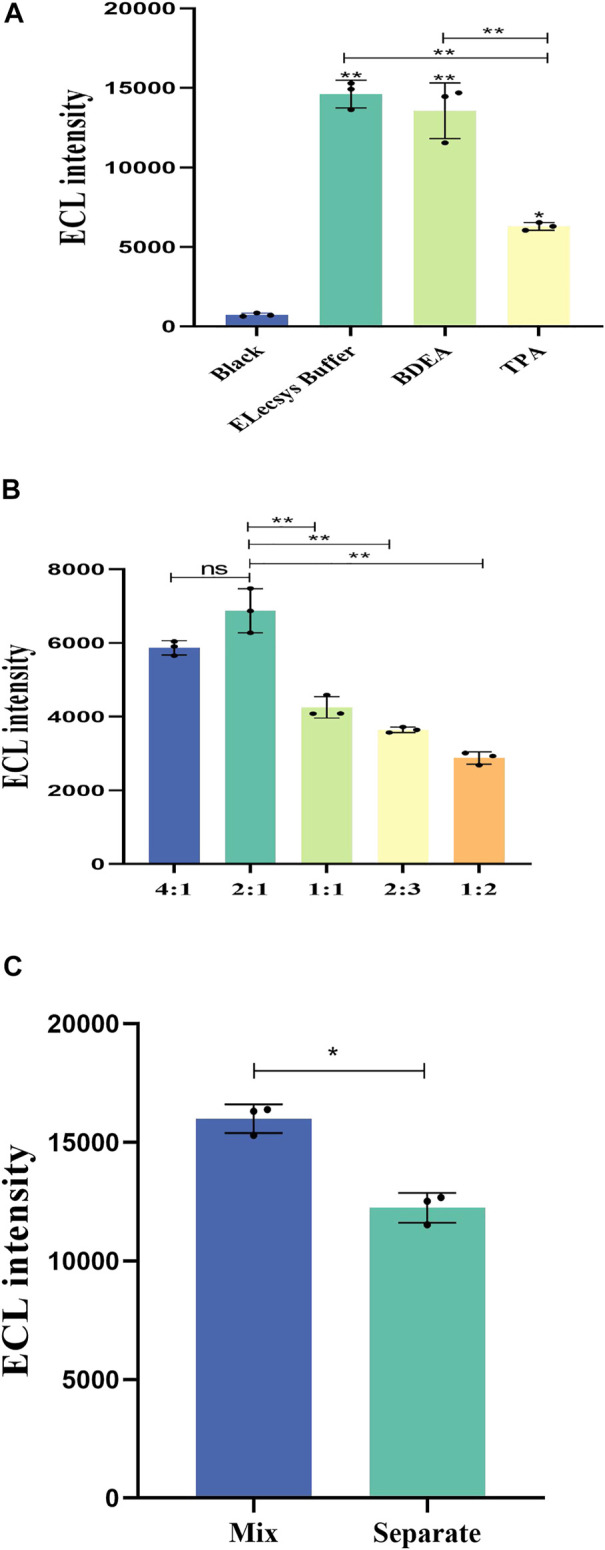
**(A)** Influence of the different co-reactants on the ECL intensity. The concentration of IFN-γ is 10 ng/mL. **(B)** Effect of different proportions of the volume of Elecsys buffer on the ECL intensity of the sample. The concentration of IFN-γ is 5,000 pg/mL. **(C)** Difference among methods of adding co-reactants. The concentration of IFN-γ is 10 ng/mL *p* < 0.05 was considered significant; A, *p* < 0.01; B, *p* < 0.01; C, *p* < 0.05.

Figure 3A shows the ECL intensity result of different co-reactants during antibody labeling. The average ECL intensity of Black, Elecsys buffer, BDEA, and TPA were 732.3, 14,613, 13,565.7, and 6,297, respectively (n = 3). Compared with the blank, BDEA, and TPA groups, the ECL intensity of Elecsys buffer was higher by a factor of 19.95,1.08, and 2.32, respectively (*p* < 0.01).

Figure 3B shows the ECL intensity of different volumes of Elecsys buffer. The average ECL intensity for Sample: Elecsys buffer ratios of 4:1, 2:1, 1:1, 2:3, and 1:2 are 5,869, 6,875.3, 4,252.7, 3,643.3, and 2,878.3 respectively (n = 3). When the volume ratio of Sample: Elecsys buffer was 2:1, the ECL intensity was significantly higher than for the other groups (*p* < 0.01).

Figure 3C shows the difference among methods of adding co-reactants. “Mix” means buffer solution and sample mixed in advance for detection. “Separate” means buffer solution and sample dropped into the sample hole sequentially. The average ECL intensity of Mix and Separate are 15,993.7 and 12,235.7, respectively (n = 3). In summary, mixing samples and Elecsys buffer in advance is better than dropping them separately (*p* < 0.05).

### 3.3 Optimization of the paper-based test

**FIGURE 4:**
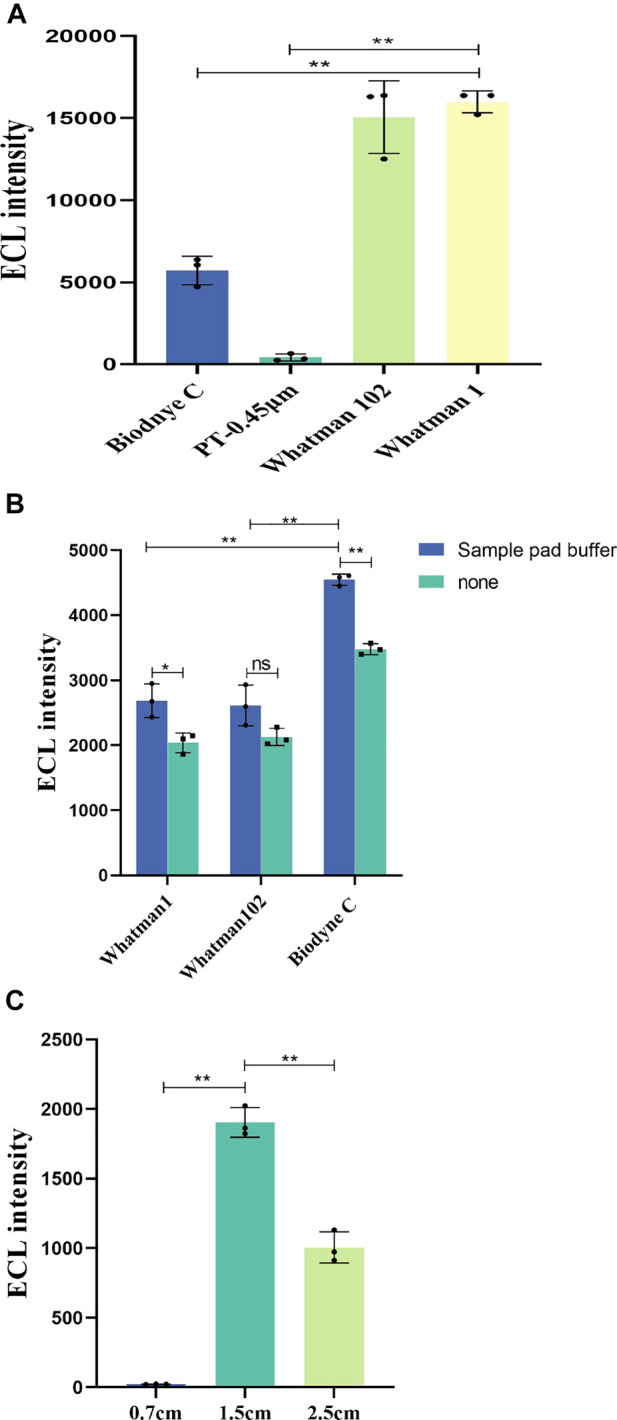
**(A)** ECL characteristics of different papers. The concentration of labeled antibodies is 0.2 mg/mL. **(B)** Effect of sample pad buffer on ECL intensity and ECL intensity of different detection pads in immunochromatography. The concentration of IFN-γ is 5000 pg/mL. **(C)** ECL intensity for different detection pad lengths. The concentration of IFN-γ is 200 pg/mL *p* < 0.05 was considered significant; A, *p* < 0.01; B, *p* < 0.01; C, *p* < 0.01.

The ECL performance for Whatman No. One and Whatman No. 102 filter media, Biodyne C, and PT-0.45 μm was tested. A 6-μL mixture of Elecsys buffer and 0.2 mg/mL labeled antibodies was dropped on each paper, and the ECL intensity was detected. The results in Figure 4A present the ECL characteristics for the different papers. The average ECL intensity of Biodyne C, PT-0.45 μm, Whatman No. 102, and Whatman No. One were 5,713, 411.7, 15,068, and 15,993.7, respectively (n = 3). The ECL intensity of Whatman No. One was higher than that of any other paper (*p* < 0.01).

Once the sample pad was treated by sample pad buffer, the treated sample pads were assembled in the paper-based channels with detection pads made of Whatman No. 1, Whatman No. 102, and Biodyne C, and the ECL intensity was measured under the same conditions for each paper. The sample pad buffer can increase the hydrophilicity of the paper and the pretreatment of samples, balance the pH of the sample to be tested, and adjust the salt ion strength. Figure 4B shows the effect of sample pad buffer on ECL intensity. The average ECL intensity of sample pad buffer group and “none” group for Whatman No. One are 2,686 and 2035.7; for Whatman No. 102 they are 2,613 and 2,127; and for Biodyne C they are 4,546 and 3,479.7, respectively (n = 3). The ECL intensity is higher after the sample pad buffer treatment (*p* < 0.01).

The results in Figure 4B show the ECL intensity for different detection pads in immunochromatography. Through experimental comparison, Biodyne C film exhibited higher ECL strength and more suitability for detection in the ECL-LFI test. Although the ECL characteristics of Whatman No. One and Whatman No. 102 are better than those of Biodyne C, in this work it is also necessary to consider the protein adsorption capacity, porosity, and wettability of the paper, as well as the capillary flow of the water-based samples. The low porosity and high protein adsorption capacity of Biodyne C are more conducive to immunochromatography. Therefore, Biodyne C was finally selected as the paper for the detection pad.

Figure 4C shows the influence of different lengths of test pads on ECL intensity. The average ECL intensity for test pad lengths of 0.7, 1.5, and 2.5 cm are 22.1, 1903.3, and 1,005.3 respectively (n = 3). Since the detection pad is a circle with a diameter of 6 mm, we set up three groups of detection pads with different lengths (0.7 cm, 1.5 cm, and 2.5 cm) for comparison. The results show that the 1.5-cm length is more suitable for the proposed ECL-LFI test (*p* < 0.01). No significant ECL intensity was found in the 0.7-cm group. The reason is that the detection pad should cover the electrodes completely, and it also should overlap the front and rear combination pads and water absorption pad by a small amount. Since the 0.7-cm detection pad length is too short, the normal flow measurement and chromatography process cannot be completed.

### 3.4 Effect of the concentration of labeled antibodies and captured antibodies on the ECL intensity

**FIGURE 5:**
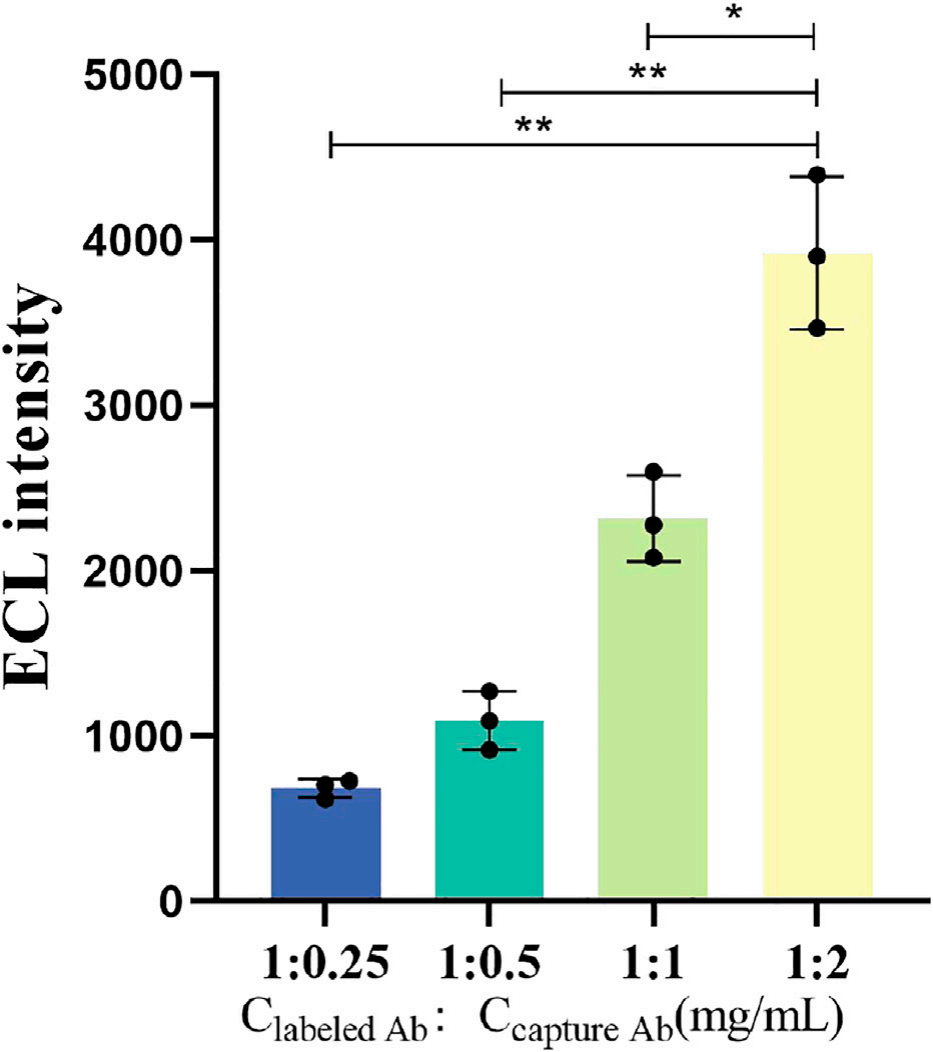
ECL intensity when the labeled antibodies reacts with the captured antibodies at different concentrations. The concentration of IFN-γ is 5000 pg/mL *p* < 0.05 was considered significant; *p* < 0.01.

To obtain higher ECL intensity, we set four groups of labeled antibodies to capture antibodies concentration ratios as follows for the ECL intensity test: 1 mg/mL:0.25 mg/mL, 1 mg/mL:0.5 mg/mL, 1 mg/mL:1 mg/mL, and 1 mg/mL:2 mg/mL. All were joined to 150 μL 1,000 pg/mL samples. Figure 5 illustrates the ECL intensity of labeled antibodies and captured antibodies with different concentration ratios. The average ECL intensity for concentration ratios of 1:0.25, 1:0.5, 1:1, and 1:2 are 684.3, 1,093.7, 2,318, and 3,921, respectively (n = 3). When the concentrations of labeled antibodies and captured antibodies were 1 mg/mL and 2 mg/mL, respectively, the corresponding signal was better (*p* < 0.01). Considering the cost of the paper-based ECL-LFI test, no more experiments were conducted on other concentrations.

### 3.5 Analytical performance of the paper-based ECL-LFI test

**FIGURE 6:**
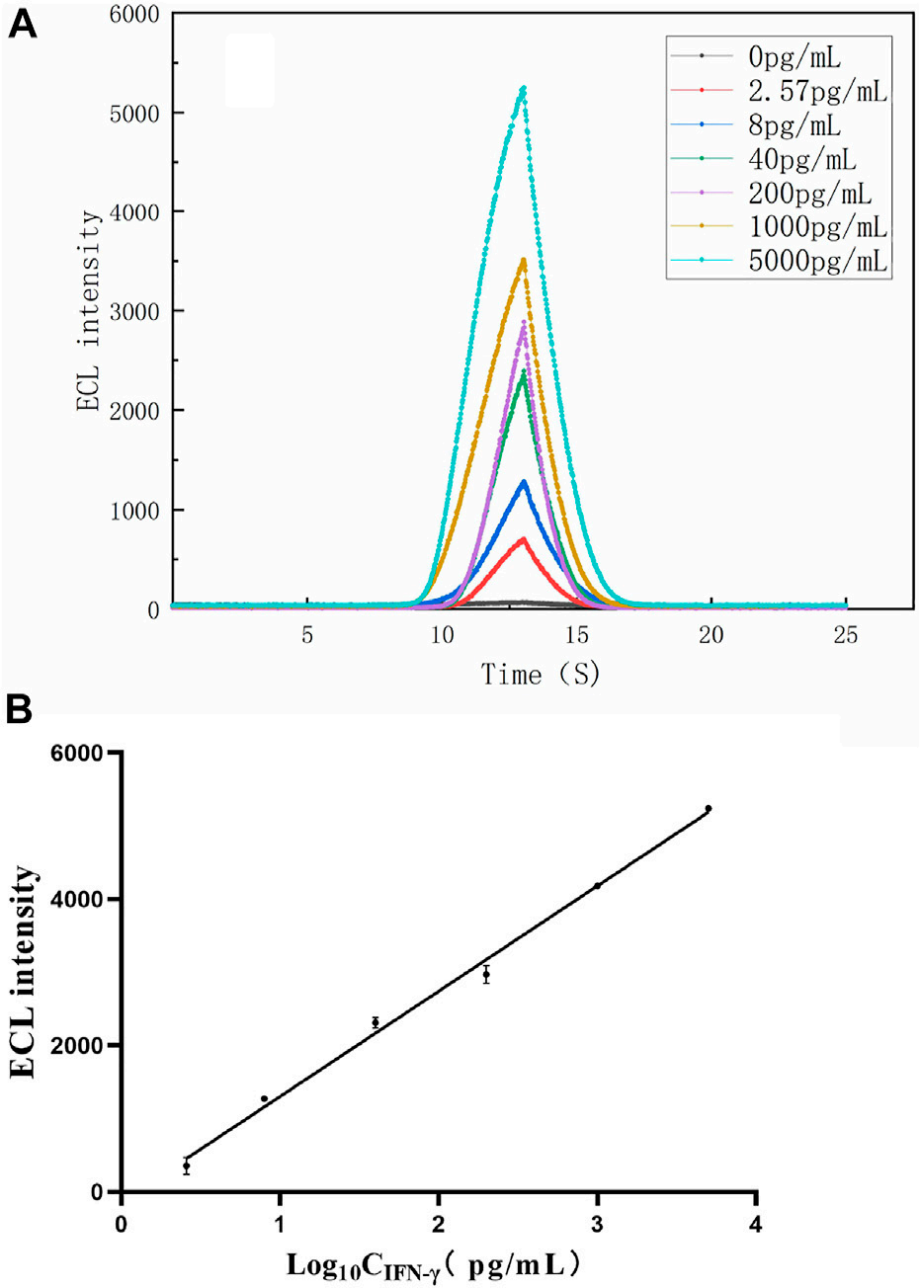
**(A)** ECL intensity-time curves of the paper-based ECL-LFI with different concentrations of IFN-γ **(B)** Linear relationship between the ECL intensity and IFN-γ concentrations of 2.57, 8, 40, 200, 1,000, and 5,000 pg/mL.

The ECL-LFI test was used to quantitatively detect the content of IFN-γ-Ag, as shown in Figures 6A, B. With increasing Ag concentration, the ECL intensity increases gradually. The calibration plots exhibited a good linear relationship between the ECL intensity and the logarithm of IFN-γ concentrations in the range of 2.57–5,000 pg/mL. The linear regression equations were IECL = 1439lg CIFN-γ-Ag − 135.5, with a correlation coefficient *R* ^2^ = 0.9932. Moreover, the detection limit for IFN-γ was 2.57 pg/mL (S/N = 3) according to the Guidelines of the Clinical and Laboratory Standards Institute (NCCLS) (Satlin et al., 2020). The results show that the paper-based ECL-LFI test can quantitatively detect IFN-γ during one potential scanning and exhibited wide linear ranges and low detection limits, indicating that it has significant value in improving the accuracy and reliability of TB infection diagnosis.

### 3.6 Analysis of IFN-γ in human serum

The reliability of the paper-based ECL-LFI test was verified by using a commercial ELISA test. Upon adding serum of unknown concentration, ECL-LFI found a result of 206.5 ± 17.6 pg/mL; the ELISA result was 201.3 ± 10.2 pg/mL. Table 1 indicates that the detection results of the ECL-LFI test for IFN-γ in human sera agreed closely with those from the ELISA test. Moreover, the recovery of IFN-γ was 106.3%. Relative standard deviation (RSD) was 5.6% for IFN-γ (n = 3). ELISA is often used alone or in combination with other methods to detect TB (Mungmunpuntipatip and Wiwnaitkit, 2020). These results show that the paper-based ECL-LFI test can reliably determine IFN-γ in human serum.

**TABLE 1:** Results of the detection of IFN-γ in human serum.

Sample	Added (pg/mL)	Found (pg/mL, n = 3)	Recovery (%)	RSD (%, n = 3)	ELISA (pg/mL, n = 3)
Serum + IFN-γ	0	7.6 ± 1.2			6.1 ± 1.0
	200	206.5 ± 17.6	103.2	8.5	201.3 ± 10.2
	1,000	1,073.2 ± 60.5	107.8	5.6	1,028.6 ± 43.5
	5,000	5,412.9 ± 310.2	108.3	5.7	5,309.2 ± 198.5

### 3.7 Specificity, reproducibility, and stability of the paper-based ECL-LFI test

**FIGURE 7:**
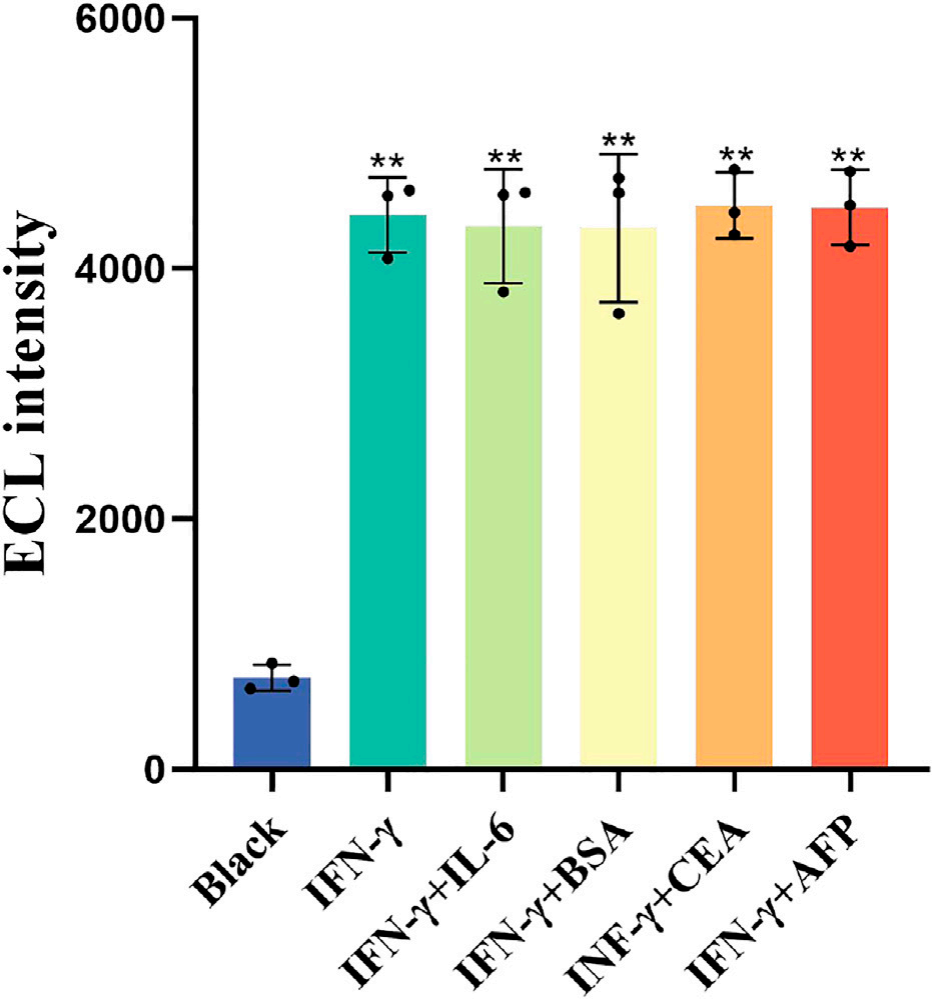
Specificity of paper-based ECL-LFI test. *P* < 0.05 was considered significant; *p* < 0.01.

The as-prepared ECL-LFI test was investigated to evaluate its specificity, reproducibility, and stability. Figure 7 shows the influence of external interference on ECL intensity. The average ECL intensity of Black, IFN-γ, IL-6, BSA, CEA, and AFP were 732.3, 4,396, 4,336.7, 4,322.3, 4,504.3, and 4,488, respectively (n = 3). The results indicate that BSA, CEA, AFP, and IL6 had no significant effect on the standard solution of IFN-γ, indicating that the ECL-LFI had a satisfactory specificity (*p* < 0.01).

Ten paper-based ECL-LFI tests were used to detect 1,000 pg/mL IFN-γ. RSD for IFN-γ is 8.1%, indicating an acceptable reproducibility of the paper-based ECL-LFI (Table 2). The stability of the ECL paper-based ECL-LFI test was calculated by preparing ECL-LFI tests in three batches, with 10 pieces in each batch, and testing the ECL intensity: RSD for ECL IFN-γ is 4.97% (Table 3), in compliance with the Guidelines of the (NCCLS). These results demonstrate that the paper-based ECL-LFI test exhibited satisfactory specificity, reproducibility, and stability.

**TABLE 2:** Reproducibility of the paper-based ECL-LFI test.

Concentration	M	Sd	RSD (%)
1,000 pg/mL	4,162.3	336.9	8.1

**TABLE 3:** Stability of the paper-based ECL-LFI test.

Concentration	M Of each batch	M	Sd	RSD (%)
1,000 pg/mL	4,417.6	4,163.5	207.02	4.97
	4,162.3			
	3,910.5			

## 4 Conclusion

The paper-based ECL-LFI test we developed enabled us to use a linear channel for electrochemical detection of IFN-γ in human serum, for the first time. This development enables us to add samples at one time and detect IFN-γ concentration in human serum without other operations. The proposed paper-based ECL-LFI test provides highly sensitive ECL signals. Moreover, the commercial ELISA test results demonstrate that the paper-based ECL-LFI test is highly reliable. Therefore, the paper-based ECL-LFI provides an effective approach for detection of IFN-γ in human serum and has potential application in facilitating accurate and reliable clinical diagnosis of TB. In addition, the successful preparation of the proposed ECL-LFI test can be applied to the detection of other diseases and lays a foundation for the preparation of multiple ECL paper-based ECL-LFI tests with multiple channels and multiple detection projects in the future.

## Data Availability

The raw data supporting the conclusions of this article will be made available by the authors, without undue reservation.
